# Supplementary material for: Factors causing emergency medical care overload during heatwaves: A Delphi study
Source: PLoS One. 2023 Dec 20;18(12):e0295128. doi: 10.1371/journal.pone.0295128 (PMC10732456; doi:10.1371/journal.pone.0295128)
Supplement: S1 Table — (DOCX) [file pone.0295128.s001.docx]

**S1 Table**

First Round: 79 statements proposed by the 15 included experts to the question “*What are the factors causing emergency medical care overload during heatwaves?*”

| lack of adeguate emergency plans (prehospital emergency medicine, emergency department, hospital) |
| --- |
| reduction of hospital beds due to staff summer holidays (hospital) |
| reduced hydration capacity by the elderly (public health) ( primary care) |
| lack of an adequate home care network for the elderly and vulnerable groups (Primary care) |
| lack of adeguate information. Public health messages about the potentially significant health risks need to be dissemitaed to the entire population. Everyone is at risk and everyone must be able to understand if a person is in danger (public health) |
| Increased heat related pathology influenced by Community based factors including social, economic, behavioural, environmental and health literacy factors |
| variable immediate access to primary care |
| Increases in health risk factors such as chronic disease potentially amenable by enhanced public health strategies and enhanced primary care |
| Limited alternatives available to prehospital paramedics. Transfer to hospital is often the quickest approach when organising social support and primary care may be more efficient and effective form a whole of system perspective |
| Emergency medical system planning often does not allow for the impact of heatwaves as it would for other disasters. Thus sudden (and largely predictable increases in demand) are often not met with additional resources. Indeed, resources are often constrained as heat related illness and carer responsibilities reduce resource availability. |
| Public risk management strategies and public awareness raising by public health authorities and community leaders is often not effective. |
| no communications plan: national, regional and local governments need to plan the response in terms of communications tools (sms, chats, mails) and media involvement. When there're no plans, not appropriate accesses and mass flows can create critical impacts into the emergency services. |
| not diffused telemedicine networks for fragile categories: health services and primary care could improve these aspects |
| medical cultural aspects: temperature (high or low) is a vital parameter but not often considered as a main parameter by primary care, emergency medical service / prehospital emergency medicine, emergency department, public health department, hospital |
| family doctors: they can educate their own patients and can suggest right lifestyles before and during heatwaves |
| health services : must be able to educate about right lifestyles during heatwaves |
| Does working in the Middle East make one more susceptible to heat stroke |
| What is leading cause of heat related illnesses in the world |
| Whats the treatment difference in different types of heat related pathologies |
| Is there a set algorithm for managing heat-related pathologies |
| WHat the rate of morbidity and mortality in heat-related illness and does it depend on geography |
| Should Emergency Departments build specialized heat stroke rooms? These need highly efficient cooling air conditioning, refrigerators stocked with iced saline and crushed ice machines, and possibly ice water bathtubs. |
| Should nations mandate reporting of heat illness, given the medical importance of the condition for national preparedness and also monitoring of climate change effects? |
| Assuming that the prevalence of heat stroke and other acute heat-related illnesses is likely higher in poorer countries, should rich countries and/or the UN determine a minimum capability that they will strive to help poor countries achieve? |
| Should the WHO determine a global information dataset regarding heat-related health effects, both chronic and acute, and invest in monitoring this information in most nations on earth? |
| Should rich nations spend very large sums of money on transformational technologies to protect people, especially in poor nations, from the direct health effects of increasing temperatures? |
| What is THE most important technological challenge to be addressed re item 5 above? Examples may include: 1. Low energy-consumption air conditioning? 2. Low energy-consumption refrigeration? 3. Low-tech, cheap, mild-moderate cooling systems for dwellings and food storage systems? 4. Quick proliferation of distributed modes of ecologically efficient electricity generation? |
| Increased demand for prehospital and retrieval medical services during extreme heat. |
| Lack of access to air conditioning in homes |
| Failure of health disaster management units to communicate risk to the community |
| Exacerbation of chronic medical conditions |
| No surge capacity in prehospital or emergency department services |
| Heat illness affecting prehospital EMS clinicians |
| Lack of screening/interventions for at risk populations during primary care encounters {Primary Care] |
| Lack of social support to vulnerable populations [Public Health] |
| Increased EMS Call Volume [EMS] |
| ED Overcrowding [Emergency Medicine] |
| Staffing shortages [Public health, Primary Care, EMS, Emergency Medicine, Hospital, Social Services agencies] |
| Poverty. (Pre-hospital), The number of people requiring emergency medical care at the time. (Emergency medical service) |
| Very young and very old. (Pre-hospital) |
| Number of healthcare staff in hospital emergency department (Emergency medical service) |
| Treatment facilities and equipment available (Emergency medical service) |
| Expertise of healthcare professionals (Emergency medical service) |
| Individuals with chronic or mental health conditions are more susceptible to extreme heat. |
| Heat waves were associated with increased overall admissions in emergency department |
| Heat waves have a greater health impact among the elderly and young children |
| Access to space cooling (build environment) |
| An acute increase in visits to the Emergency Department is caused by heat effects on adults and children for a range of causes, including respiratory illness, renal disease, heat injury and accidents. The causes of these diseases which are excerbated are complex and outside the Emergency Department remit. |
| Reducing greenhouse gas emissions will be reduce future heatwaves |
| An acute increase in visits to the ED could be managed better by heatwave warnings to inform staffing and other measures by the ED. [Emergency Department] |
| Better case management in primary care of chronic diseases and frailty could avoid some emergency admissions |
| Hospital: incident command system not trained to scenario heatwave specific on long period |
| ED lack of communication inside chain of care and digitalisation( IT overview tools ) |
| ED : high complexity of diseases and treatment on heatwave ED have not good organisation, Stuff, Staff, for clinical decisions units and response protocols |
| Poor primary care and poor private health preparedness at home , focus on vulnerable groups like elderly, children , not mobile patients at home ( dehydration, medication) . Preclinic treatment insufficient for response after 16:00 and organised treatment at home |
| EMS dispatch Center initial Actions goals mostly to transportation to ED No physician inside dispatch Center |
| ED : no protocols not enough space or stuff for short term treatment in heatwave situations |
| limited resilience of. the elderly (pre end in) |
| limited preparedness in organization of sports events (pre , disaster) |
| higher risk of drowning (pre) |
| unawareness of risks of physical activity (pre and in - hospital) |
| heat stroke (pre hospital) |
| dehydration (pre and in hospital) |
| Inadequate identification of high risk groups and preparations to minimise the effects of a heat wave in these groups. |
| Inadequate public health messaging of the risks of heatwaves |
| Insufficient preparedness by the community for heatwave events, with impacts at the ED. |
| Inadequate training of GPs and ED staff on signs, symptoms and management of heat stress |
| Inappropriate public events planning that occurs in peak periods for heat stress |
| Limited community knowledge of the signs and symptoms of heat illness from general practitioner and public health sources |
| preventive measures are better than treating a patient with hyperthermia |
| A social network prevents to become a victim of a heat wave |
| Heat waves have a severe influence in the afternoon |
| Heat waves have no influence on hospitalized patients. |
| Heatwaves coincide with vacation periods, when medical staff is taking annual leave - complete healthcare sector |
| Insufficient means and attention for heatwaves - political level |
| Understaffing of healthcare sector due to long-term sick leave after COVID-19 crisis - complete healthcare sector |
| Overburdened healthcare sector with patients experiencing other morbidities (e.g. COVID-19) - complete healthcare sector |
| Insufficient preventive measures before and during heatwaves - population and public health level |
| Increasing number of vulnerable (elderly and comorbid) people in the population - population level |
