# Supplementary material for: Factors causing emergency medical care overload during heatwaves: A Delphi study
Source: PLoS One. 2023 Dec 20;18(12):e0295128. doi: 10.1371/journal.pone.0295128 (PMC10732456; doi:10.1371/journal.pone.0295128)
Supplement: S2 Table — (DOCX) [file pone.0295128.s002.docx]

**S2 Table: Included Experts’ Characteristics**

Country

| Australia | 4 |
| --- | --- |
| Belgium | 3 |
| Italy | 2 |
| India | 1 |
| Israel | 1 |
| Greece | 1 |
| UK | 1 |
| USA | 1 |
| United Arab Emirates | 1 |

Area(s) of expertise

| Public Health alone | 3 |
| --- | --- |
| Emergency Medicine + Disaster Medicine | 10 |
| Emergency Medicine + Public Health | 1 |
| Disaster Medicine + Public Health | 1 |
