# Supplementary material for: Factors causing emergency medical care overload during heatwaves: A Delphi study
Source: PLoS One. 2023 Dec 20;18(12):e0295128. doi: 10.1371/journal.pone.0295128 (PMC10732456; doi:10.1371/journal.pone.0295128)
Supplement: Attachment — Submitted filename: Response to reviewers.docx [file pone.0295128.s003.docx]

**Manuscript No. PONE-D-23-26567**

*“Factors Causing Emergency Medical Care Overload During Heatwaves: A Delphi Study” s*ubmitted to PLOS ONE.

We thank the Reviewers for highlighting the strengths of our paper; however, some concerns have been raised, and these are discussed below.

| **Reviewer #1**  The authors conducted a Delphi study to derive the causes of emergency medical care overloading during heatwaves. Based on the analysis of the consensus 18 statements, the main factors are around patient processing, identifying stakeholders, the population, primary care, as well as dedicated resources and bed capacity. My recommendation is major revision given my following comments | **Answers** |
| --- | --- |
| As a research study, evaluation/validation of the results is an indispensable section. However, the authors claim that the evaluation is their next step. From my point of view, evaluation/validation should be added to make this study solid and compact. | We agree with the reviewer that evaluation and validation of study results are fundamental in research. However, we also believe that the present manuscript should not contain such sections at this stage for several reasons, which will be explained below – some concepts, probably already known to the expert reviewer, will be expressed only to clarify our answer better.  First, the peculiarity of Delphi Studies methodology is to collect and organize the information obtained to be used in further discussions (such as focus groups or panel meetings) aiming at solving problems in fields in which knowledge is poor or conflicting. Such discussions have completely different methods to be applied and constitute different studies, despite being connected by the same overarching aim.  In fact, this Delphi study stems from a project aiming at “*Mitigating the overload caused by heatwaves on emergency medical care*” which for now achieved a preliminary scoping review phase (confirming the scarce evidence in the field; see Paganini et al, 2023 10.1097/MEJ.0000000000000975), and the present Delphi Study. This project entails a next step to disseminate the Delphi Study results via formal publication and talks at congresses, attract the interest of scientific societies (national or international), and start discussions on how to create and implement focused interventions in EDs and EMSs.  We could consider this as a stand-alone study containing all the elements of a scientific study, despite being not quantitative: a solid method – consistent to seminal literature produced in this field –, detailed results, and a discussion of the key findings. Previous Delphi studies have been published in this form, without the following discussion / implementation / verification phase, since these are actually separate studies. Also, it is not infrequent to read qualitative studies presenting, for example, clinical findings to be further validated in other contexts before implementation in guidelines.  Finally, climate change is an issue to be urgently addressed by the scientific community. We believe that waiting for the results of shared discussion, or implementation and verification of this Delphi’s findings will dramatically delay dissemination of results to other researchers. This study is reporting not only statements reaching agreement, but also those who did not, to provide a potentially more complete information to the scientific community. It is true that the conclusions of this study are valid only for the expert panel and not automatically for all the emergency care systems; other healthcare systems could perceive a statement which did not reach consensus as important according to their specific features or priorities. Especially in fields such as public health, healthcare systems and emergency care resilience (which is currently overstretched and in need of solutions), the complete results (positive and negative) could provide useful insight.  For all these reasons we believe that the study should be published in this form. |
| The Conclusion section is weak since it just repeats the contents in the abstract. Please revise it to present a more comprehensive and stronger summary. | Thanks for the suggestion. We improved the Conclusion section by first giving the reader a summary and insight of the importance of the problem, then we summarized some of the actions proposed by experts to mitigate heatwaves impact on emergency care. |
| The Discussion section is comprehensive, but the presentations need improvements. From the current version, it is hard to efficiently grasp the core findings and insights. This section should be more systematic. | As we initially followed the input-throughput-output model for patient flow in the ED and EMS to discuss the results, we clarified this approach and re-organized this section into these three phases – using clear subheadings – sections to improve comprehension and readability. |
| In Table 1, the mean values seem to reflect the significance of statements. Are there any insights from the mean metric? | We added a few comments on means (see: *lines 117-118 and 154-155*). Further comments were not deemed important, nor associations were noted, since values were very close between each other. |
| Based on the mostly focused aspects authors highlighted, Table 1 could be better if a column of statements' corresponding aspects (i.e. identifying stakeholders, the population, primary care, etc.) is added. In this way, each statement can be directed to the associated aspect. | Thanks for this suggestion. Table 1 was modified by adding a column clarifying the section of patient flow and the target of potential intervention, also mirroring the discussion. |
| **Reviewer #2**: | **Answers** |
| It would be valuable to frame a limitation section that explicitly addresses the geographical bias, given that a majority of the experts participating in this Delphi study are from high-income countries. | Thanks, we inserted a “Limitations” subheading, which is already highlighting the issue of experts’ country of origin. |

On behalf of all co-authors,

Matteo Paganini
